# Supplementary material for: Competitive Effects of Oxidation and Quantum Confinement on Modulation of the Photophysical Properties of Metallic-Phase Tungsten Dichalcogenide Quantum Dots
Source: Nanomaterials (Basel). 2023 Jul 15;13(14):2075. doi: 10.3390/nano13142075 (PMC10385026; doi:10.3390/nano13142075)
Supplement: Supplementary file 1 [file nanomaterials-13-02075-s001.zip › nanomaterials-2475859-supplementary.pdf]

Supplementary Materials:

Competitive Effects of Oxidation and Quantum Confinement on Modulation of the Photophysical Properties of Metallic-Phase Tungsten Dichalcogenide Quantum Dots

Figure S1. AFM images of WX2-mQDs

Figure S1 shows AFM images of WX2-mQDs-*S* (a, c) and WX2-mQDs-*L* (b, d) on a mica substrate. The insets of each figures are the histogram of size distribution.

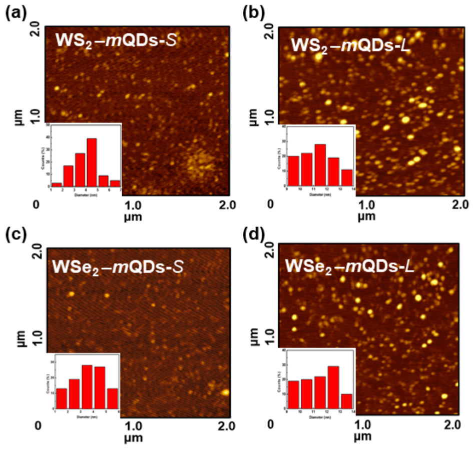

Figure S2. XPS spectra of WX2-mQDs

XPS peak comparison of tungsten core level (W4f) measured from the metallic phase WX2-mQDs. The spectra are displayed by stacking plot.

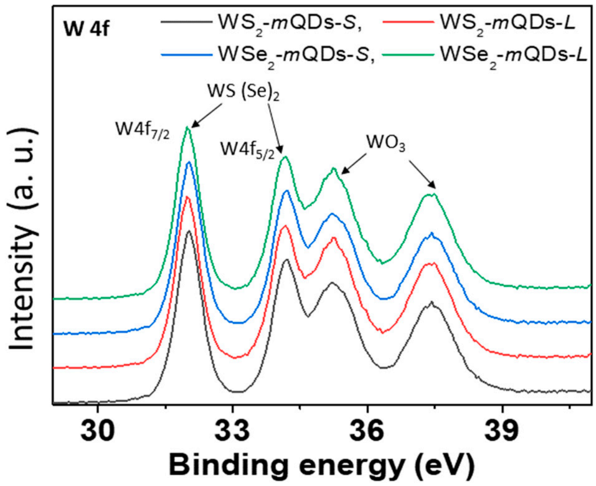

Figure S3. Zeta potential of WX2-mQDs

As the material dispersed in solution often carry electrical charges, zeta potentials of the WX2-mQDs were measured. The zeta potential of WX2-mQDs are all negative, however, WX2-mQDs-*L* have -20.2 (-20) mV of zeta potential bigger than -5.6 (-8.9) mV of WX2-mQDs-*S*. The negative surface charge should make the dispersion stability of WX2-mQDs.

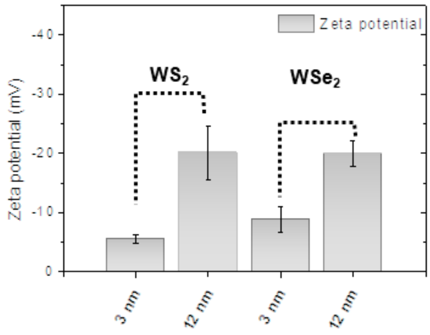

Figure S4. UV-Vis absorption spectroscopy of WS_2-mQDs-*S* (a) and WSe_2-mQDs-*S* (b)

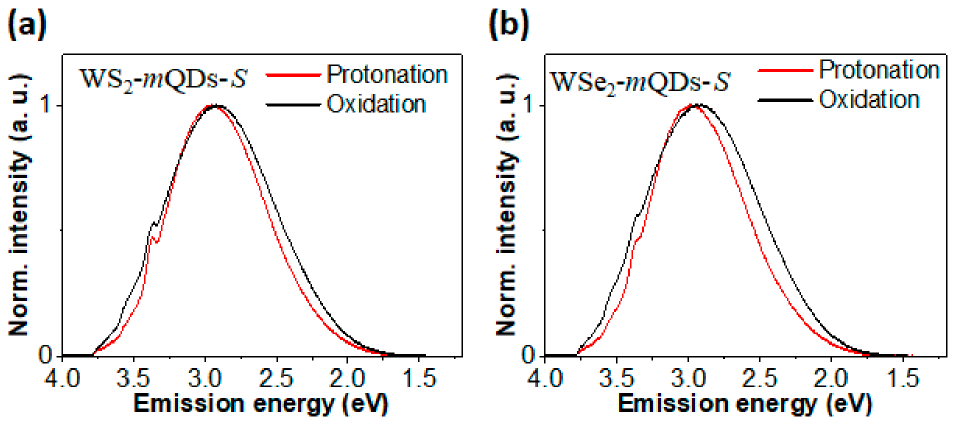

Figure S5. UV-Vis absorption spectroscopy of WS_2-mQDs-*L* (a) and WSe_2-mQDs-*L* (b)

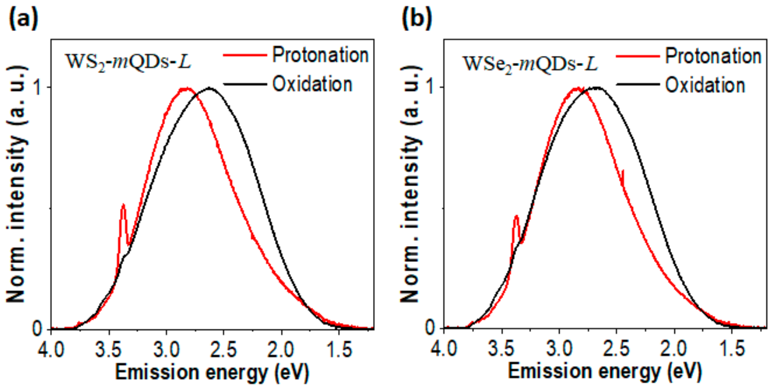

Figure S6. TRPL of WX2-mQDs

Figure S5 show the intensity normalized PL spectra with different integrated time measured from WS_2-*m*QDs-*S* (a), WS_2-*m*QDs-*L* (b), WSe_2-*m*QDs-*S* (c), and WSe_2-*m*QDs-*L* (d), respectively. The black, red, and blue lines are the integrated PL spectra for 0-2 ns, 2-6 ns, 6-12 ns, 12-20 ns, 20-30 ns and 30-42 ns, respectively.

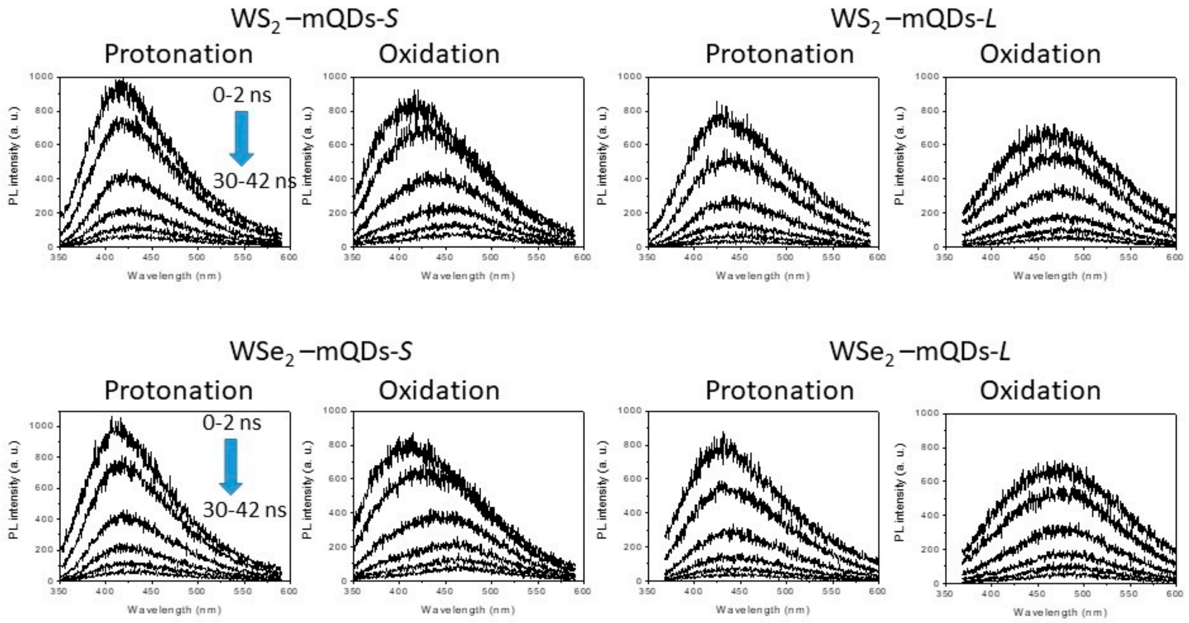

Figure S7. Lifetime of WX2-mQDs.

Figure S6 shows the time-resolved PL decay profile of protonated and oxidized WX2-mQDs. The PL lifetimes are calculated from the fitting of a multi-exponential function, $y = a_1 e^{-x/t_1} + a_2 e^{-x/t_2} + a_3 e^{-x/t_3} + y_0$. The average PL lifetimes are estimated by the following equation[5] ; $t_{avg} = \sum_i A_i \times t_i^2 \,/\, \sum_i A_i \times t_1$

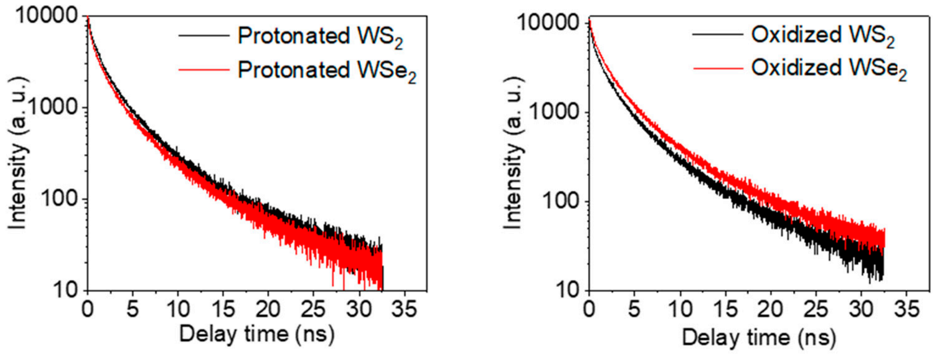

References

1. Song, S. H.; Kim, B. H.; Choe, D. H.; Kim, J.; Kim, D. C.; Lee, D. J.; Kim, J. M.; Chang, K. J.; Jeon, S. *Advanced Materials* **2015,** 27, (20), 3152-3158.
2. Kim, B.-H.; Jang, M.-H.; Yoon, H.; Kim, H. J.; Cho, Y.-H.; Jeon, S.; Song, S.-H. *NPG Asia Materials* **2021,** 13, (1), 1-9.
3. Park, K. H.; Yang, J. Y.; Jung, S.; Ko, B. M.; Song, G.; Hong, S.-J.; Kim, N. C.; Lee, D.; Song, S. H. *Nanomaterials* **2022,** 12, (10), 1645.
